# Developing cessation interventions for the social and community service setting: A qualitative study of barriers to quitting among disadvantaged Australian smokers

**DOI:** 10.1186/1471-2458-11-493

**Published:** 2011-06-24

**Authors:** Jamie Bryant, Billie Bonevski, Christine Paul, Jon O'Brien, Wendy Oakes

**Affiliations:** 1 Centre for Health Research and Psycho-oncology, Cancer Council New South Wales, Priority Research Centre for Health Behaviour, University of Newcastle, Hunter Medical Research Institute. Room 230A, Level 2, David Maddison Building, Callaghan, NSW, 2308, Australia; 2 Health Behaviour Research Group, Priority Research Centre for Health Behaviour, School of Medicine & Public Health, University of Newcastle, Hunter Medical Research Institute. Room 268 Level 2, David Maddison Building, Callaghan NSW 2308, Australia; 3 Cancer Council NSW. 153 Dowling Street, Woolloomooloo, NSW, 2011, Australia

**Keywords:** Smoking, Vulnerable Populations, Inequalities, PRECEDE-PROCEED model

## Abstract

**Background:**

Smoking rates remain unacceptably high among individuals who are socially disadvantaged. Social and community service organisations (SCSO) are increasingly interested in providing smoking cessation support to clients, however little is known about the best way to assist disadvantaged smokers to quit in this setting. This study aimed to explore barriers and facilitators to quitting within the conceptual framework of the PRECEDE model to identify possible interventions appropriate to the social and community service setting.

**Methods:**

Semi-structured focus groups were conducted with clients attending five community welfare organisations located in New South Wales, Australia. Thirty-two clients participated in six focus groups. A discussion guide was used to explore the barriers and facilitators to smoking and smoking cessation including: current smoking behaviour, motivation to quit, past quit attempts, barriers to quitting and preferences for cessation support. Focus groups were audio-taped, transcribed and analysed using thematic analysis techniques.

**Results:**

Participants were current smokers and most expressed a desire to quit. Factors predisposing continued smoking included perceived benefits of smoking for stress relief, doubting of ability to quit, fear of gaining weight, and poor knowledge and scepticism about available quit support. The high cost of nicotine replacement therapy was a barrier to its use. Continual exposure to smoking in personal relationships and in the community reinforced smoking. Participants expressed a strong preference for personalised quit support.

**Conclusions:**

Disadvantaged smokers in Australia express a desire to quit smoking, but find quitting difficult for a number of reasons. SCSOs may have a role in providing information about the availability of quit support, engaging disadvantaged smokers with available quit support, and providing personalised, ongoing support.

## Background

According to the World Health Organisation, tobacco is the single greatest preventable cause of death and disease worldwide [1]. It is a leading risk factor in the development of chronic diseases including cancer, lung diseases, and cardiovascular disease and is responsible for more than 5 million deaths each year [1]. If current trends continue, the number of deaths caused as a result of tobacco is expected to rise to between 8 and 10 million deaths annually by 2030 [2-4]. Within Australia, tobacco is estimated to be responsible for 7.8% of the total burden of disease [5], and costs the economy more than $31.5 billion dollars each year [6].

Public health campaigns, tobacco control programs and tobacco control policies have resulted in significant declines in the prevalence of tobacco use in many developed countries in recent decades [7-9]. Currently, prevalence of daily smoking in Australia is 16.6%, declining more than 30% since 1991[10]. However despite this overall decline, smoking rates remain unacceptably high among those who are both socially excluded and socioeconomically disadvantaged. For example, smoking rates are markedly higher among low income single women (46% [11]), individuals with a mental illness (41-62% [12,13]), and the homeless (66-77% [14-17]).

Although disadvantaged smokers attempt to quit at rates similar to other smokers [18], they are less likely to succeed [18-21]. Social and community service organisations (SCSOs) are emerging as a novel and viable setting for targeting socially disadvantaged and marginalised groups for smoking cessation [22-24]. SCSO provide welfare services to socially disadvantaged individuals across a broad range of areas including support in accessing accommodation, emergency relief (groceries, assistance with paying bills), financial and relationship counselling, family support and support for individuals with a mental illness. SCSO are increasingly aware of the contribution of tobacco use to social exclusion, poverty and health disparities, and are interested in developing interventions addressing smoking cessation among their clients [25].

Developing effective interventions for novel settings requires thorough formative research to determine the normative beliefs and perceived barriers to change among the population to be targeted, and ensure a culturally relevant and acceptable intervention is developed [26,27]. A considerable amount of research has explored barriers to quitting smoking, including among specific disadvantaged sub-groups (those living in socioeconomically deprived areas, institutionalised public mental health patients [28], and pregnant Aboriginal and Torres Strait Islander women [29]). Barriers including poor self efficacy, lack of knowledge, lack of willpower, pro-smoking community norms and barriers to accessing support are frequently identified [30-33]. However health behaviours are embedded within a social and cultural context [34], which is especially important to consider when attempting to address health disparities in vulnerable or marginalised groups [35]. A limited amount of research has explored barriers to cessation among disadvantaged Australian smokers, identifying stress as a barrier to quitting, and resilience as an important factor for quitting and maintaining abstinence [28,36-38]. However, no research has explored barriers to quitting among severely disadvantaged individuals accessing community service organisations, nor examined these factors within a conceptual framework to identify appropriate individual-level intervention strategies appropriate to the community service setting [39].

The PRECEDE model [40] is a particularly valuable and widely applied framework for guiding the development of interventions [41]. Within the PRECEDE framework, factors contributing to health behaviours are classified as those that predispose, enable and reinforce behaviour. Predisposing factors are antecedents to behaviour including attitudes, knowledge, beliefs and self-efficacy for change. Enabling factors are those that help facilitate behaviour change such as availability of resources. Reinforcing factors include rewards, social support and attitudes of significant others that facilitate and reward change [42]. The PRECEDE model has been used extensively to guide planning of health behaviour interventions [41] including developing smoking cessation interventions to increase the provision of quit smoking counselling by primary care physicians [43], and has been applied to changing other health behaviours in disadvantaged groups including routine cancer screening and prevention of ischemic heart disease through changes to smoking, diet, and physical activity [44,45]. The utility of the PRECEDE model is its capacity to consider in a systematic way the factors that influence health behaviours. This in turn allows identification and implementation of appropriate and effective strategies for behaviour change [39].

This study sought to describe the smoking behaviours and attitudes of disadvantaged Australian smokers attending SCSOs, including past experiences of quitting, preferences for quit support, and perceived barriers to quitting. These perceptions and experiences were considered within the conceptual framework of the PRECEDE model to provide recommendations for the development of appropriate individual-level interventions in the social and community welfare setting.

## Method

### Design

As part of a study examining the acceptability of the SCSO setting for providing smoking cessation support, semi-structured focus groups were conducted with clients attending five non-government community organisations for welfare support. Focus groups are integral to developing and tailoring complex interventions to address individual needs in different settings [46], and are well suited to in-depth exploration and understanding of underlying issues embedded within a social context [35].

### Sampling

Chief Executive Officers (CEO) of community service organisations in New South Wales, Australia, were approached for permission for their organisation to participate in a study examining smoking and quitting among disadvantaged clients. Community social service organisations are non-government organisations that provide welfare services to individuals in need in the communities in which they are based. Purposeful sampling was used to ensure inclusion of a diverse range of service and client types [47]. Following verbal or written consent, CEOs nominated services within their organisation to participate. Co-ordinators of services were briefed about the study and asked to distribute study information and consent statements to eligible clients. Clients who were in contact with the community service organisation and self reported smoking tobacco were eligible to participate in a one hour focus group. Sampling continued until both facilitators agreed that saturation had been reached and that no new insights or themes were identified by participants [48,49].

### Procedure

Focus groups were conducted between December 2008 and March 2009 by two facilitators, one with training in behavioural science (JB), and one with experience working in the community service sector (JO). Each focus group was conducted at the participating community organisation in a private room. Prior to commencement of the research, participants were given an information statement and consent form and also had information about the study explained verbally. Participants were informed that the discussion would be audio-taped, but that only de-identified quotes would be used in reports arising from the research. Participants provided written informed consent prior to the commencement of discussions and were provided with a $50 gift voucher for reimbursement of their time and travel costs. The study gained ethics approval from the University of Newcastle Human Research Ethics Committee. Each participating community service organisation also provided approval for involvement of their organisation.

### Discussion Guide

A semi-structured focus group protocol was used to guide discussions. Focus group questions were developed by the research team based on a review of the literature and consideration of the key research questions. Questions were designed to explore the barriers and facilitators to smoking and smoking cessation. Participants were asked about their current smoking behaviour (type of tobacco used, number of cigarettes used each day, times when they smoke more or less) and current motivation to quit. The focus groups allowed participants the opportunity to detail past quit attempts, including the type of help or support used, and what facilitated or undermined each quit attempt. Participants were asked about their preferences for cessation support, including whether they would like help to quit, perceptions of the role of the community organisation in providing support, and details about specific types of support they would or would not like to receive.

### Analysis

Discussions were audio-taped and transcribed verbatim. All transcripts were checked by the first author (JB) for typographical errors. Transcripts were analysed using thematic analysis techniques by reviewing each transcript and noting emergent themes. To establish reliability and validity of emergent themes, two transcripts were independently analysed by both facilitators (JB and JO) and identified themes compared and reconciled with input from the second author (BB) where necessary [50]. Analysis of the remaining transcripts was conducted by JB using Nvivo version 8. The following results are presented thematically, with barriers to quitting considered within the context of the PRECEDE model. De-identified quotes presented in subsequent analysis are followed by parentheses which describe the service the client attended (A-F: see Table 1) and the gender (Male or Female) of the speaker.

**Table 1:** Focus group participant number and gender by service type.

Service	Total *N*	Female *N*
Service A: Child, youth and family early intervention	5	5
Service B: Community care centre	6	2
Service C: Residential drug and alcohol program	8	8
Service D: Residential adolescent life management service	3	0
Service E: Infants and child services	6	5
Service F: Outreach service for homeless youth	4	2

TOTAL PARTICIPANTS	32	22

## Results

### Participant and Group Characteristics

Six services from within five community organisations participated. Details of service and participant involvement are presented in table one. Participating services included two early intervention services for teenage mothers, one residential youth drug and alcohol rehabilitation service, one adult residential drug and alcohol rehabilitation service, one outreach service for homeless youth, and one community care drop in service that provided counselling and crisis relief services. Thirty-two clients, 22 female and 10 male, participated in six separate focus groups. Other demographic characteristics were not collected as individual-level and subgroup comparisons were not the aim of this study. All participants were aged over 16 years. Focus groups lasted between thirty-four minutes and one hour (*M*= 50.33 minutes), and comprised between 3 and 8 participants. All participants were current daily or occasional smokers and were either attending the community service organisation or had attended in the past.

### Smoking behaviour

Most participants reported initiating smoking in their early teen years. One client reported starting smoking at the age of five or six years. The main reasons for initiating smoking included to fit in with friends and having brothers, sisters, and parents who smoked. About one third of participants reported smoking between 10 and 15 cigarettes per day, and a similar proportion reported smoking between 15 and 20 cigarettes per day or smoking one pack or more per day. Participants reported that the amount they smoked increased remarkably when they were socialising with friends and family who were also smokers and when drinking alcohol. The majority of participants seemed heavily addicted to smoking, reflected by most participants reporting that they smoked their first cigarette soon after waking or even that they woke up during the night to smoke. Participants perceived themselves as highly addicted, describing smoking as having "*a hold on me" (E, Female) *and being "*part of my life now" (E, Female)*.

Most participants reported multiple past attempts to quit smoking. Many reported trying to quit cold turkey without support or use of cessation aids such as NRT. NRT had been used by some participants, but were generally considered ineffective. One participant said: *"I have used all sorts of things, patches, the nicotine gum....They don't work" (F, Male)*. Three clients reported that they had tried Vaerenecline with some success "*Last year I was taking Champix [Varenecline].... Yeah, they were really good. Um, I gave up for 10 weeks and I wasn't cranky or anything" (C, Female)*. Several participants reported contacting the Quitline, but few perceived the support offered useful "*I rang them ages ago, but it didn't really do anything" (D, Male)*.

### Barriers and facilitators to quitting smoking

Barriers to quitting smoking identified by participants were analysed thematically then categorised as those predisposing, enabling and reinforcing continued smoking.

#### Predisposing Factors

##### Strong motivation to quit

The majority of participants reported a strong desire to quit smoking. Short and long term health benefits like feeling fitter, being healthier and a fear of smoking related diseases like emphysema and lung cancer were the main reasons given for wanting to quit. *"I've quit many times. I'm at the point now nearly that I'm going to quit for good. I feel as though I'm sick of all me mates dying around me because of lung cancer" (F, Male)*. The high cost of smoking was another strong motivating factor with participants reporting that finding money to smoke was a continual source of stress given their low incomes. *"It gets pretty hard after a while thinking 'how am I going to get my next pack of durries(cigarettes)'? Or when you run out it's like, what do I do, how am I going to get my next lot of money to get them?" (F, Male)*.

##### Beliefs in the benefits of smoking for stress relief

Although the financial and health consequences of smoking were well understood by participants, many participants held a strong belief that smoking had many benefits. Smoking was described as relaxing, calming, a good way to relieve boredom and a "*best friend*" and a "*superglue*" that could hold a person together during stressful times. One participant said *"I need it to help me stress-less and yeah, take my mind off a lot of things" (D, Male)*. Many participants used stress as a strong justification for continuing to smoke. *"I need to stop.... But at the moment I'm very stressed out so I don't think I should stop at the moment. It does help me with stress relief heaps" (B, Female*). The use of smoking as a form of stress relief was also a commonly cited reason for relapse *"I gave it away and then 7^th ^of July last year, went off for four months and then me nerves played up on me so I went back on" (B, Male)*

##### Doubting ability to quit

Despite a strong reported desire to quit smoking, many participants expressed doubt in their ability to successfully quit *"I would like to quit but I honestly, I know this sounds bad, I honestly don't think I have the will power to do it. I honestly don't think I do" (E, Female)*. Participants descried quitting as "*impossible*" and the idea of making a quit attempt was often intimidating "*I know I want to quit - it's just hard to do. I'm scared to do it" (A, Female)*. Feeling 'ready' and having willpower to quit were identified as the key to success *"I think you've got to be ready aswell-you've got to want to feel ready within yourself. I know that's hard to say, 'well when are you going to be ready to actually want to do it?' You've got to think hard about it" (A, Female)*.

##### Poor knowledge of available quit support

Participants overall knowledge about the availability of quit support was poor. Many participants who had used NRT reported that it did not effectively reduce cravings, but often reported not wearing patches as prescribed, not using recommended doses of gum, and were unaware of recommendations to use stronger doses of NRT or multiple forms if they were heavy smokers. Several participants reported being told by others that NRT is ineffective, and this perception had discouraged some from using NRT during a quit attempt. One participant said: "*I've been told that those stupid Nicorette patches don't work and the gum's gross and it doesn't work so, there's no point in even wasting your money on buying them if they're not going to help you" (A, Female)*. The majority of participants had no knowledge about what Varenicline was, how to access it or the cost. Knowledge of other support services such as the telephone quit service Quitline was also poor. While many participants had heard of Quitline, which is heavily advertised on television, many were unsure about the type of support Quitline provided, including the provision of the call-back service or that the service is free.

##### Fear of gaining weight

Among many female participants, fear of gaining weight was also a barrier to making a quit attempt. Participants recounted stories about friends and family members who had given up smoking and then gained weight, or reported that they had experienced weight gain themselves during previous quit attempts *"I gave up for 5 months last year and gained about 40 kilos. Um yeah, and just took it back up again" (C, Female)*. One participant who had recently started smoking after a long period of abstinence reported loosing ten kilos when she began smoking again, which she described as "*a nice side effect" (B, Female)*.

#### Enabling Factors

##### Limited provision of cessation support

Some participants had received advice from their General Practitioner (GP) about the use of Bupropion or Varenicline, but most were unaware that prescription only cessation medications were available through their GP. Some clients reported '*being told' *or lectured by their GP to quit smoking without the offer of support to quit "*Most doctors just tell me 'it's bad for your health, you've got to stop. I advise you to quit"(A, Female)*. Young mothers who had recently had repeated contact with physicians during prenatal and antenatal care reported being given educational pamphlets and advice to stop smoking, but felt they were not offered genuine support or assistance to quit *"Yep, that's the most they give you. A pamphlet" (A, Female)*. As a result most reported that they continued to smoke throughout their pregnancies.

##### Limited use of available quit support

Despite awareness of the existence of the telephone Quitline, only three clients reported having contacted Quitline in the past. There was strong scepticism among participants that support provided over the telephone would be useful in aiding a quit attempt. Young participants were particularly doubtful about the motivations and ability of a person who did not know them personally helping them to quit smoking. The following two quotes illustrate this point - *"It's a bit weird talking to some random person, you're like, oh yeah I want to quit and you know what I mean? They might not really care - they're just doing it for a job." (D, Male). "Nup. Wouldn't want to waste my time. Because they're getting paid to give you useful advice and they're not really supportive"(D, Male)*.

##### High cost of NRT

NRT was perceived as an expensive and ineffective substitute for smoking that would require a large initial outlay of money *"I've looked at the patches occasionally and thought I'm not paying $32 or $35 for a box. It's just too expensive" (B, Female)*. Because of doubts about the effectiveness of NRT many participants did not recognise that if they were successful at quitting smoking, NRT would not be an ongoing cost "*If they don't work then it's a waste of $50"*. When asked, the majority of participants agreed that if NRT was free or available at a heavily subsidised rate that they would consider using NRT *"I'd take it for sure.... If you said patches they are for free or $2.50, I'm telling you there would be way more people having a crack at giving up" (E, Female). "Subsidise the quit smoking products.... maybe someone could subsidise these products so that they're affordable" (C, Female)*.

#### Reinforcing Factors

##### Smoking and Social Norms

Repeated social and environmental exposure to smoking was also a barrier to quitting smoking for many participants. Smoking was reported as a normal part of social interaction, with participants stating that the majority of their partners, family and friends also smoked *"you've got your family and your friends come over and they're like oh yeah, and they light up...."(A, Female); "You always know someone that smokes" (A, Female)*. Participants spoke about smoking being depicted on television, seeing people smoking when walking down the street and commented that "*you seem them everywhere you go" (A, Female)*. Not only did this strong presence of smoking in the community make it less likely for participants to make a quit attempt, it also served as a powerful trigger for relapse "*Yeah, given up about 20 times in that time but yeah, for some reason just don't work because everyone else around me smokes and it's hard to quit" (F, Male)*. One participant reported being strongly motivated to quit and had tried setting quit dates in the past, but found quitting impossible because of the continued exposure to second-hand smoke at home *"Well I have been trying to give it up. I sort of set today as a give up target, but I'm going to find it so hard because people are smoking outside my room at home" (B, Male)*. Several participants mentioned changing social norms around smoking, such as restrictions on smoking at shopping centres and at pubs often, made them feel '*uncomfortable' *and '*ashamed' *of their smoking, however no participants identified this as a factor motivating them to quit.

### Preferences for Quit Support

When asked about the type of support they would like to receive to quit smoking, participants emphasised the need for personalised, ongoing support. *"Support... I don't know, just a social worker to come around and you know, just have a bit of a chat...meet them at the park or something" (A, Female)*. Several participants emphasised the importance of having someone who genuinely cared about them providing support to quit *"I'd like to go to someone for some serious advice, you know, someone who actually cares and will support you (D, Male) "Yeah, someone you can talk to and you're not going to talk to once and then they're not going to be there again.(D, Male)*. Family and friends who often were also smokers were considered a poor source of support.

## Discussion

This qualitative study extends knowledge of barriers to quitting smoking by examining barriers and facilitators among disadvantaged smokers attending SCSOs in Australia. Identifying factors that predispose, enable and reinforce a particular behaviour within the framework of the PRECEDE model provides a basis for the development of appropriate interventions to specifically target barriers to behaviour change.

While most participants reported a strong desire to quit smoking and had made multiple past quit attempts, predisposing factors acting as barriers to quitting included using smoking as a way of coping with stress, poor self efficacy, and fear of gaining weight. These findings confirm individual level barriers to quitting smoking identified among disadvantaged smokers both in Australia [36-38] and the UK [31,32,51], and particularly highlight the perceived role of stress and coping in continuing to smoke [31,32,38,52], and the perception of willpower as the key to successfully quitting [32].

Poor knowledge about and low utilisation of available quit support were reported across the focus groups. Few participants reported ever receiving help to quit smoking from their GP and few had called the Quitline, which seemed to stem from a lack of understanding about the type of support offered. Despite Varenecline being available in Australia as a prescription-only smoking cessation treatment since January 2008 at a minimal cost for low income smokers [53,54], few participants knew that this support was available or had accessed it. While participants had good knowledge of the availability of NRT, there were misconceptions about its use and effectiveness, and the cost was perceived as prohibitive. The availability of free or subsidised NRT was strongly supported. Participants strongly articulated a preference for ongoing, personalised support.

The predisposing, enabling and reinforcing factors identified suggest that strategies to increase knowledge of and engagement with evidence-based smoking cessation strategies may be crucial to overcoming barriers to quitting for disadvantaged smokers. Access to services is recognised as an important barrier for smokers attempting to quit in lower socioeconomic groups [30,55]. Integration of referral and direct provision of smoking cessation support into the SCSO setting may also hold significant potential in addressing key barriers identified by SCSO clients. SCSOs are increasingly interested in addressing aspects of physical health that impact on wellbeing, and are well placed to provide cessation support given that they are heavily utilised by disadvantaged smokers (there are more than 5,700 SCSO in Australia [22,23]). Recent research has noted the acceptability of providing support in this setting [22-24]. Interventions provided in this setting should focus on enhancing client access to existing services including Quitline and subsidized pharmacotherapy, and address individual barriers to quitting through integration of brief advice as part of usual care. A large randomized controlled trial to examine the effectiveness of providing brief advice, access to NRT and referral in the SCSO setting is planned [56].

### Study strengths and weaknesses

A number of limitations regarding recruitment and sampling should be considered when interpreting the results of this study. While care was taken to recruit a range of organisations offering a variety of services to a cross-section of disadvantaged individuals, as a result of our sampling approach our findings are indicative only of the opinions of disadvantaged smokers who access community social services. Secondly, potential bias in the inclusion of organisations and clients should be considered. While the majority of services contacted agreed to take part, it may have been that only those services interested in smoking cessation agreed to their clients being contacted as part of the study. We did not collect detailed demographic information from participants. Furthermore, clients were recruited by staff of community service organisations with no involvement from researchers, which may have resulted in the selection only of clients known to be interested in smoking cessation. Finally, although the PRECEDE theory was chosen a priori to explore data, the researchers were cautious not to impose bias on data analysis. All themes emerged from the data and were not pre-determined by the theory. As a result of using this framework, which is behavioural in nature, structural barriers to quitting may not have been identified.

## Conclusions

This is the first study to explore smoking behaviours, past quit attempts and barriers to quitting among disadvantaged smokers attending community service organisations for welfare support in Australia. Our findings identify multiple complex barriers to quitting, but suggest that SCSOs may have a role in increasing knowledge and use of available cessation support, and providing direct, personalised, and ongoing support to disadvantaged Australian smokers. Further research is needed to explore the effectiveness of these approaches.

## Competing interests

The authors declare that they have no competing interests.

## Authors' contributions

All authors conceived of the study and participated in study design and co-ordination. JB co-facilitated the focus groups, analysed the data and drafted the manuscript. JO co-facilitated focus groups and assisted with data analysis and drafting of the manuscript. BB, CP and WO assisted in drafting of the manuscript. All authors have read and approved the final manuscript.

## Pre-publication history

The pre-publication history for this paper can be accessed here:

http://www.biomedcentral.com/1471-2458/11/493/prepub
